# Advanced cancer palliative care economics in sub-Saharan Africa: an important start

**DOI:** 10.1016/S2214-109X(21)00486-1

**Published:** 2021-10-29

**Authors:** Walter Ochieng

**Affiliations:** Office of the Director, Center for Global Health, US Centers for Disease Control and Prevention, Atlanta, GA 30329, USA

The International Agency for Research on Cancer estimates that there were 811 000 new cancer cases and 534 000 cancer deaths in sub-Saharan Africa in 2018, and cancer cases are rising.^1^ Many people with cancer present late for care, leading to high out-of-pocket payments and concomitant risk of household financial ruin. This burden has resulted in calls for governments to fund comprehensive cancer care, including palliative care, within universal health care schemes.^2^ These calls are occurring while countries in sub-Saharan Africa grapple with competing decisions around equitable and efficient implementation of universal health services under increasingly constrained resources and emerging threats, including COVID-19.

Decisions on new investments are fraught with uncertainty about their effects on health systems, households, and individuals. There are risks in displacing resources from existing programmes to fund new ones. Local evidence can help to guide adoption of interventions. Bates and colleagues^3^ provide relevant evidence in their timely and pioneering prospective study on the economics of advanced cancer palliative care in Africa published in the *Lancet Global Health*.

The approach and the data collection tool (Patient and Carer Cancer Cost Survey) developed and used by the authors offer other researchers a useful template for conducting similar studies in other sub-Saharan Africa countries.^3^ Bates and colleagues endeavored to address two major gaps in previous economic evaluations of palliative care programmes: spillover and distributional effects.^3–5^ The modified societal perspective they adopted allowed them to assess the spillover effects of a nascent palliative care programme in Malawi on households by explicitly assessing changes in productivity and disutility in informal care givers. The survey tool allowed them to collect both direct and indirect health-care costs and the prospective design mitigates recall bias.

The authors found that palliative care might be associated with a 22% lower household catastrophic health expenditure and dissavings (nine [47%] of 19 households who received palliative care *vs* 48 [69%] of 70 households who did not [relative risk 0·69, 95% CI 0·42–1·14, p=0·109]). By assessing dissavings (when households sell assets or borrow money to pay for health care) as an integral component and consequence of catastrophic health expenditure, the authors draw attention to the interrelationships between adverse health, household vulnerability, intergenerational poverty, and worsening inequalities. They found that dissavings and catastrophic expenditures were three times higher in the relatively poorer households that did not receive palliative care compared with the wealthier ones that did.

Evidence from the early days of HIV programmes suggested that antiretroviral services were initially regressive—with benefits mainly accruing to higher educated, wealthier, urban-dwelling patients who could access and successfully navigate large hospital-based programmes, before trickling down to poorer, patients living in rural areas.^6^ The current study suggests that this might already be the case with extant palliative care programmes in Africa. Policy makers should be wary of inadvertently worsening wealth inequalities if palliative care programmes are limited to urban hospitals. Successes in broadening access that have been achieved in other programmes including task shifting, task sharing, and telehealth, could be used in the scale-up of palliative care services.

The authors needed to bound their analyses due to sparse data and to make their models tractable. The authors restricted their analytical time horizon to 6 months, which is insufficient to capture longer-term benefits of palliative care on other household economic measures, like childhood education and welfare^4^ or increased costs accruing from palliative care-related positive survival benefits.^7^ By restricting the analyses to households where the patients survived, they omitted funeral costs, which could outweigh direct household treatment costs in parts of sub-Saharan Africa.^8^ These important findings should therefore be viewed as likely underestimates of the potential impact of palliative care.

Although their study was limited to a narrow range of advanced cancers, their approach can be extended to other terminal medical conditions. The authors’ call for future studies assessing structural barriers surrounding the integration of palliative care within existing programmes and for newer tools to capture its broader benefits are therefore timely. Experience suggests that the scale and rate of dissavings often evolve with progressing disease severity, concomitant escalating medical treatments, and declining household productivity. Households might be more likely to dispose of an asset that is easily exchanged for money, such as an animal, before taking usurious loans or selling land. Studies looking at the effect of early initiation of palliative care on dissavings would be useful.

There is a Kenyan saying that serious health-related suffering is like termites eating through a hut—the vulnerabilities it causes are not always apparent. The work of Bates and colleagues should be extended and the initial evidence they have generated could influence ongoing discussions related to palliative care and cancer care in sub-Saharan Africa.
